# On the Use of Design Thinking: A Survey of the Brazilian Agile Software Development Community

**DOI:** 10.1007/978-3-030-49392-9_5

**Published:** 2020-05-06

**Authors:** Matheus Prestes, Rafael Parizi, Sabrina Marczak, Tayana Conte

**Affiliations:** 6 University of Oslo, Oslo, Norway; 7 Monash University, Clayton, VIC Australia; 8 IT University of Copenhagen, Copenhagen, Denmark; 9 University of British Columbia, Vancouver, BC Canada; 10 MunDDoS Research Group – School of Technology, Pontifícia Universidade do Rio Grande do Sul (PUCRS), Porto Alegre, Brazil; 11 Instituto de Computação, Universidade Federal do Amazonas (UFAM), Manaus, Brazil; 12 Instituto Federal de Educação, Ciência e Tecnologia Farroupilha (IFFAR), São Borja, Brazil

**Keywords:** Agile, Design Thinking, Industry professionals, Survey

## Abstract

Design Thinking (DT) has been chosen as an approach to support problem-solving by many software development companies. However, there are divergences between the professionals of these companies concerning which techniques are performed, which steps are followed, and the way to implement this approach, as it proposes itself, to be divergent to generate numerous alternatives and, also, convergent, to find a solution. For this reason, aiming to characterize how the software companies have been implemented DT, this paper presents the results of a survey answered by 127 professionals from the Brazilian software industry. The results report a variety of scenarios in which DT has been applied: more than ten different models (sets of steps) are followed by the professionals; more than 50 techniques have been used, mainly, for meeting the needs in the process, according to the context of use and based on previous experiences. We also present 29 computational tools that, according to the respondents, assist the execution of DT, in addition to the integration with agile methods, allowing them to generate ideas and solutions, to explore and understand the problem.

## Introduction

Design Thinking (DT) seeks to solve problems through design principles, exploring possible user needs and validating solution proposals through prototypes. It is used in software development to foster creativity and innovation in the generation of new features and products, as well as DT has been chosen as an approach to problem-solving by many software development companies [1, 2].

By bringing the user needs to the center, DT also improves team communication and facilitates knowledge domain acquisition, which are well-known issues in software development [3]. Given its interactive and dynamic nature, DT is considered an easy-in integration and a way to boost agile development [4]. While the focus of DT is on the creation of various prototypes identify the better solution, Agile methods are concerned with uncertainties and risks at the beginning of the development process, seeking to develop software incrementally, delivering the product as soon as possible [5]. Using DT integrated with Agile methods fosters a better alignment of the expectations of both customers and developers. Also, this integration helps to gather the needs of customers in the early stages of software development, ensuring the usability of the software [6].

DT have a flexible structure according to the company’s business logic [7]. Therefore, it is important to understand if there is a script to be followed when DT is applied to software development contexts like process model to be followed, techniques to support the model steps, tools, artifacts, and roles involved.

Literature contains gaps about how industry professionals have made use of DT in software development. Thus, we executed a survey to identify which models and techniques and tools the professionals are using, reasons for choosing DT, usage scenarios, and the benefits and difficulties of applying DT.

The main contributions of this papers are (i) discuss about the use of DT into software project, summarizing which techniques are most used, what models, phases, and steps are performed to understand the user’s necessities and to create innovative software, and; (ii) know the integration of DT in agile methods by professionals in the Brazilian software development industry.

This paper is structured as follows: Sect. 2 shows earlier studies about Design Thinking and agile software development; Sect. 3 presents the methodology we conducted to achieve our goals, describing in details how we had performed the survey; Sect. 4 exposes the outcomes after the survey application and the results’ discussion. Finally, in Sect. 5 we conclude our research showing future actions to gathering new and relevant results.

## Earlier Studies on Agile and Design Thinking

DT is used to create innovative projects for human-centered design [8]. As a property of DT, we have a multidisciplinary strategy, with techniques and practices that can be applied to many types of project, as well as focused on satisfying the expectations of users of the product/service developed based on its structure [9]. Brown (2008) [8] also reports that DT fits the use of designers empathy to address what is technologically suitable and feasible when proposing a solution.

Considering the integration between Agile and DT, there is a vast literature in the field of Software Engineering, since DT allows the search for a solution oriented to meet the user’s needs, while agile methods are strongly collaborative, focused on characteristics such as speed, simplicity, continuous and fast deliveries, frequent feedback collection and quick reaction to changes [10–17].

Rhinow and Meinel [18] present an empirical study to evaluate the integration of DT in large corporations with frameworks such as Lean and Scrum, consulting the expectations of project managers through 50 interviews. The results pointed that DT fosters teamwork associating value with deliveries and the continuous improvement of the process, aligned with the philosophy of Lean. In relation to development projects, managers realize that DT encourages the inclusion of visual representation of the need (prototype), the definition of a business model and a complete definition of the activities necessary to produce the appropriate solution (user story map).

Nedeltcheva and Shoikova [19] presented a study about DT combined with Scrum claiming that DT helps to understand what needs to be done, while Scrum gives autonomy to decide how to do it. They also argue that DT and Scrum are similar because both are iterative, requiring adopters to develop sufficient insights to recognize initial successes and failures through constant evaluation and adaptation. The study present a set of advantages of the integration of both methodologies, such as help to create products or services which meet the current user needs, and that organizations can reduce risks from the development achieving better results for their efforts.

Prasad et al. [16] attempted to answer how to apply DT practices to improve customer expectations in Agile process. They conducted 15 interviews with industry professionals from organizations in Sri-Lanka, resulting in a set of best practices, which were classified into five areas, such as (a) customer’s real need identification; (b) transforming customer’s real needs into pilot solutions, (c) visualizing the pilot solution for customer feedback; (d) idea generation for the pilot solution, and; (e) brainstorming. As a result of the research, they proposed a framework as a way to help organizations enhance customer satisfaction using design thinking practices in agile practices, involving activities that comprise the five major defined areas.

The study of Darrin and Devereux [5] discusses the impacts of the application of Agile and DT principles in systems development processes. Mapping Design Thinking, Agile Manifesto and System Engineering, the authors report that these approaches act to more actively incorporating the users in the whole product and process development, including some practical implications such as a better customer engagement with the team; the requirements releases would result as an iterative process; and the process in an iterative way provides the generation of multiple design and implementation options, supporting the agility and reducing risks and uncertainties.

Pereira and Russo [6] present a literature review to evaluate how DT is integrated with Agile methodologies, selecting 29 studies which report that the integration of these approaches is applied throughout the development cycle, being the Scrum the most commonly Agile method used. Also, the integration between Design Thinking and Agile has shown that the customers are satisfied with the products developed and their needs are fulfilled, as well as there is an improvement of usability, supporting the proper management of challenges or requirements discovering.

Our work reports a study that seeks to characterize how professionals in the software development community, based on Agile methods, use DT in their processes, presenting which techniques, models, phases, and steps are performed. Therefore, our research provides an overview of the use of DT, going beyond the works already presented in the literature.

## Research Setting

Looking for answers to know how industry professionals have used Design Thinking, we developed a survey to seek a more in-depth understanding of the Brazilian software development community. The survey developed in this work is characterized as explanatory [20, 21], seeking understanding of the phenomenon through the information collected.

In this section, we start presenting how we carried out the planning and design of the survey, proceeding with it’s prior validation, and after we describe details of the execution. In Sect. 4 we show the outcomes gathered with our survey, discussing the findings.

### Planning, Design and Prior Validation

We built this survey as a mechanism aimed at deepening the knowledge about the use of DT by the industry. We started to build the questionnaire containing 11 questions as a data collection instrument, using the Qualtrics^1^ tool. Table 1 shows the questionnaire structure, where the respondents initially answer questions related to DT, such as DT methods, techniques, and tools (Block 1), and purposes, contexts, benefits and difficulties to using DT (Block 2). Finally, we questioned the professionals about their jobs, in order to draw a profile of the respondents (Block 3). The questions of the survey were created based on data gathered previously through a systematic literature mapping.

**Table 1.** Questionnaire structure

#	Question	Type
Block 1		
1	There are several process models, which abstract workspaces when using Design Thinking. Do you use any of these models as a reference in your activities?	Closed
2	Several techniques can be used to support the use of Design Thinking. What techniques do you usually use?	Closed
3	How do you usually decide which techniques to use?	Closed
4	On a scale of 0 (No difficulty) to 10 (Extreme difficulty), how difficult do you feel in deciding which techniques to use in a given situation?	Closed
5	Do you use any software (or computer system, as you prefer to call it) to support the use of Design Thinking techniques?	Closed
Block 2		
6	For what purpose do you use Design Thinking in software development?	Closed
7	What are the common usage scenarios where you use Design Thinking?	Closed
8	In your experience of using DT in software development, what would you point out as benefits or positives brought about by adopting the approach?	Open
9	And what would be the difficulties or the negative points?	Open
Block 3		
10	What is your experience, in years, using Design Thinking?	Closed
11	What is your current organizational role or function?	Open

Before conducting the survey distribution to the defined target audience, we performed a prior validation process. Following the recommendations given by Kitchenham [22] about empirical research, a pilot test was performed for evaluate the consistency and correctness of our survey.

### Execution

Following the survey’s planning and design process, we defined the target audience, who should be professionals working with DT in the software development process. We define as a strategy to reach out to such professionals and to electronically distribute the questionnaire, the use of the professional-oriented social network, the *LinkedIn*^2^.

In *LinkedIn* we apply filters to find the professionals who serve the target audience, according to the strings: *“design thinking” and “software”* and *“design thinking”*, filtering by Brazilian nationality.

## Results

The survey’s period ranged from September 2019 to December 2019. During this time, the survey request was sent to 466 professionals, resulting in 149 participants, of which 127 answered the questionnaire until the end. The response rate was 31,97%. The “n” is variable because not all questions were required, so some may contain fewer answers. As shown in Table 1, we have divided the structure of the questionnaire into 3 blocks. To present the profile of the respondents, we first describe about the background information of them.

### The Respondents’ Profiles

Respondents were asked about their experience in years of using DT. Table 2 illustrates the professional’s experience organized in absolute and percentage values. The largest number of respondents (60 respondents = 47,24%) reported having between 1 and 3 years of experience using DT. Considering those with more than 4 years of experience, we reach to 39 respondents (30,71%).

**Table 2.** Years of experience

Answer	n	(%)
Less than 1	28	(22,05)
1–3	60	(47,24)
4–7	32	(25,20)
More than 7	7	(05,51)
Total	127	100%

They were also asked about their position in organization. Most consider themselves a Agile Coach, with a total of 18 answers (14,17%), the second as a UX/UI Designer (17 respondents = 13,39%), and in third place as a Facilitator (16 respondents = 12,60%), as shown in Table 3. Also, 30 respondents (23,62%) pointed out the option “Other”. These subset of professionals includes positions such as Product Managers, Development Coordinators, and Process Analysts. This result shows how expressive it is to professionals’ positions and the use of DT in software development.

**Table 3.** Respondents’ position in organization

Position	n	(%)	Position	n	(%)
Agile Coach	18	(14,17)	Analist	5	(3,94)
UX/UI Designer	17	(13,39)	Engineer	5	(3,94)
Facilitator	16	(12,60)	Developer	5	(3,94)
Product Owner	12	(9,45)	Researcher	1	(0,79)
Expert	10	(7,87)	Other?	30	(23,62)
Consultant	8	(6,30)	Total	127	100%

After knowing the profile of the professionals who answered the survey, knowing their level of experience on the subject, and their use of DT in their activities, we did an individual analyze of each question presented in the questionnaire, starting with the questions about DT methods, techniques, and tools.

### DT Models, Techniques, and Tools

Respondents were asked about which models they follow in the application of DT. Table 4 shows the results, and in this question, it was possible to choose more than one model, because we consider that more than one model can be used by an organization, even in the same project. The proposed models were extracted from the literature [23].

The four models that were chosen by more than 10% of respondents were: (i) Divergent and Convergent (93 respondents = 72,44%); (ii) Stanford d.school (72 respondents = 56,69%); (iii) Stanford d.School integrated with Hasso Plattner Institute (HPI) (58 respondents = 45,67%), and; (iv) Hasso Plattner Institute (HPI) (47 respondents = 37,01%). On the option “other”, were mentioned the Stanford d.School model integrated with Convergence and Divergence; Double Diamond; Massachusetts Institute of Technology (MIT) approach; and a model created by the respondent’s own company.

**Table 4.** Models used by the respondent’s

Model	n	(%)	Model	n	(%)
Divergent Convergent [24]	93	(72,44)	Meinel and Leifer [25]	13	(10,24)
Stanford d.School [26]	72	(56,69)	HCAW** [27]	11	(8,66)
Stanford d.School + HPI [28]	58	(45,67)	Diving board [29]	10	(7,87)
HPI* [30]	47	(37,01)	Sandino [31]	8	(6,30)
Brown [32]	30	(23,62)	Other	6	(4,72)
Nordstrom [33]	22	(17,32)	I don’t know	3	(2,63)
IBM Model [34]	19	(14,96)	Total	391	

* Hasso Plattner Institute

** Human Centered Agile Workflow

We also asked what techniques are commonly used during DT application sessions. To do so, we presented 46 techniques that we brought from the literature and even allowed new techniques to be mentioned, if they existed. Table 5 presents the top 10 techniques most chosen by respondents, the three most selected being Brainstorming (119 respondents = 88,15%); Personas (118 respondents = 87,41%), and; Empathy Maps (97 respondents = 71,85%).

**Table 5.** 10 most chosen techniques

Technique	n	(%)
Brainstorming	119	(88,15)
Personas	118	(87,41)
Empathy Maps	97	(71,85)
Costumer Journey Maps	94	(69,63)
Business Model Canvas (BMC)	87	(64,44)
Interview	84	(62,22)
Storytelling	84	(62,22)
User story	83	(61,48)
Observation	81	(60,00)
Storyboard	81	(60,00)

All 46 techniques that were made available to respondents in the survey were selected by at least two of them. In addition, 11 new techniques were suggested by the participants. Thus, there are a total of 59 different techniques that provide aid to the application of DT in software development, as well as shows that there is a great variation between the techniques.

In this way, considering this wide range of techniques, we questioned the reasons that lead the professional to choose a particular technique over others (Table 6). The respondents reported that the most determining reason for choosing a particular technique is that it fits their needs (109 respondents = 85,83%); they choose according to the context in which they are working (101 respondents = 79,53%); they choose based on previous experience (99 answers = 77,95%); and the respondents choose the techniques according to DT space/step, where each space/step has its own techniques (82 answers = 64,57%).

**Table 6.** Reasons for choosing techniques

Reason of chosen	n	(%)
When the technique fits my need	109	(85,83)
It depends a lot on the context I am going to use	101	(79,53)
Based on my previous experience	99	(77,95)
I choose the techniques according to the DT space/step, where each space/step has its own techniques	82	(64,57)
Recommendation by my company	24	(18,90)
By referral from a colleague	22	(17,32)
I already have my catalog of techniques that I always use	19	(14,96)
I usually need to study the techniques because I never know which one is best for the moment	13	(10,24)
Another reason?	3	(2,36)

Having known the reasons that lead to the choice of techniques, we had questioned how difficult it is to make this choice, to make this decision. We therefore asked what is the difficulty level for the choice, ranging from 0 (slightly difficult) to 10 (extremely difficult). The result obtained for this questions was an average of 4,55 difficulty of choosing the techniques, with a standard deviation of 2,23, which indicates that there is considerable variability in terms of difficulty of choice and it is considered that It is not an easy task to do.

And, to conclude the information on DT models, techniques, and technologies, we encouraged respondents about the computational tools that support the process. Respondents presented a set of 29 different tools that help their work and application of DT techniques for different activities. Table 7 shows the computational tool set. We can conclude that there is no specific software focused on DT and its tasks, since it is a methodology composed of different actions aimed at fostering creativity.

**Table 7.** Tools used by the respondents

Tools		
Marvel app	Build	Illustrator
Paint	Canvanizer	Photoshop
Evrybo	Google Sheets	SAPBuild
Xmind-Stakeholder map	Adobe XD	Smaply
Miro	Google presentation	Strategyzer
Whimsical	POP	Axure RP
Figma	Mindmeister	Touchpoint dashboard
Mural	Invision	Creately
Real Time	Hotjar	Circle
Muraly	Survey Monkey	
Total	29 tools	

‘

### Purposes, Contexts, Benefits and Difficulties to Using DT

Here, our intention was to discover the reasons that led to the choice of adopting DT in the company’s processes, in which contexts DT has been applied, the benefits of its use, and what makes application/use of DT a difficult task.

Initially, we questioned about the reasons that lead professionals to use DT. The three most selected answers were (i) to generate ideas and solutions (120 respondents = 94,49%); (ii) to explore and understand the problem (113 respondents = 88,98%), and; (iii) to create innovative ideas, and to reduce uncertainties (both with 89 respondents = 70,08%) (Table 8). This question was multiple choice, i.e. the respondent could choose more than one answer, as DT can be applied for more than one reason (Table 8).

**Table 8.** Purposes to use DT

Purpose	n	(%)
To generate ideas and solutions	120	(94,49)
Explore and understand the problem	113	(88,98)
To create innovative ideas	89	(70,08)
To reduce uncertainties	89	(70,08)
Understand and specify requirements	84	(66,14)
Improve customer satisfaction	75	(59,06)
Bring the development team closer to the customer	72	(56,69)
Easy relationship with agile methods	57	(44,88)
Win user’s empathy	53	(41,73)
Software Validation	40	(31,50)
To manage projects	16	(12,60)
For game development	5	(3,94)
Other?	2	(1,57)
Total	815	

In addition to the purposes listed in the Table 8, DT has also been characterized for assisting agile methods, and as a mechanism for strategic planning, industrial problems, complex problems, adjustments, and process improvements.

We also explored in which scenarios DT is applied on software development. Table 9 shows the professionals understanding that DT is mostly used in multidisciplinary team scenarios (107 respondents = 84,25%); to create innovative products/software (94 respondents = 74,02%), and; used in partnership with Agile methods 92 respondents = 72,44%).

**Table 9.** Scenarios of use of DT

Scenario	n	(%)
With multidisciplinary teams	107	(84,25)
Creation of innovative products/software	94	(74,02)
Used in partnership with Agile Methods (Lean, Scrum)	92	(72,44)
Create co-creation among project participants	79	(62,20)
Innovation as a whole, from the development process to software	71	(55,91)
Changes and improvements in software development	51	(40,16)
Within a daily/weekly software development process, accompanied by the entire team (from client to developer)	39	(30,71)
Other?	6	(4,72)
Total	539	

We asked the respondents about the benefits of adopting DT in their projects. They pointed out as a benefit that DT seeks to understand the users in detail and fosters creativity.

Their answers related to benefits for the users were:

- Keep the user at the center of the process without neglecting business needs;
- Greater empathy with the user;
- Focus on customer need;
- High user collaboration;
- Understanding customer pains;
- Closeness of the technical team with the customer;
- Reach endpoint user.

Finally, we asked about the difficulties faced by professionals to apply DT in their software projects. The following quotes were cited:

- Match project to time and scope;
- Adapting people to use methods;
- Detachment of solutions (contributors already come with the solution and not with a real understanding of the problem);
- Transform qualitative data into data valid for the corporate environment;
- In evolution projects with very defined scope (such as migration/modernization), Design Thinking is not very applicable
- Projects with very defined architecture (with third party technologies or tools) also make it challenging to use DT because in these situations there is little room for innovation;
- Not always does the customer have the time to know the problem in-depth;
- Lack of experience driving the design.

### Discussion

Based on the results of the survey, there is a variety to choose DT models to follow, the techniques to be used, the software that supports the activities. Also, it is important to consider that the respondents argued they have difficulties to choose some techniques to apply in DT sessions. This highlights the issue of DT being dynamic, allowing adaptations during the course of its development, considering the profile of the participants, with the needs of the client, and with the context of carrying out the techniques.

Other important point is to consider the integration between DT and Agile methods, since 92 of the respondents answered that in their organizations DT is used integrated with Agile, indicating that approximately 3/4 of the companies represented by the participants integrate both approaches (Table 9).

Regarding the benefits of using DT, we can identify that the user is defined as the center of attention, with the development team being responsible for meeting the needs of this user, showing that the industry understands that DT in software development is an user-centered approach.

In another scenario, DT carries with it difficulties inherent in the integration and collaboration of different professionals in a multidisciplinary way. This issue is clear when we analyze the difficulties in applying DT, such as match the project to time and scope; adapting people to use methods; not always does the customers have the time to know the problem in-depth, or they think they know what is the best solution previously, among others.

## Concluding Remarks and Perspectives

This paper presents a survey to know about the use of DT in software development by industry professionals. As a result, 127 responses from professionals working in the Brazilian software development industry were registered, which allowed us to advance the literature in the field of software engineering.

The survey’s answers show the experience of the professionals, their job profiles, how they use the techniques and methods of DT. We also discovered that the most used model is the Divergent Convergent method, as well as a wide range of techniques and computational tools, in addition to those previously presented in the literature. The results indicated too that 3/4 of the companies develop DT integrated with Agile methods, considering like the main proposals to use DT: to generate ideas and solutions; to explore and understand the problem, and; to create innovative ideas.

We presented in this article the main benefits of using DT, according to the participants, including keeping the user at the center of the process without neglecting business needs; greater empathy with the user; focus on customer needs, among others. On the other hand, we listed the difficulties for the application of Design Thinking, being the most important ones, according to the participants: (i) matching project to time and scope; and (ii) preparing people to use it.

Our work presents as limitations that we cannot generalize to the entire universe of software development since we conducted the survey only in the Brazilian scenario, and answers may only represent the respondent’s view and not the whole organization of which they are part. Nevertheless, these limitations represent opportunities to replicate this survey in different countries. These replications would allow the community to build a more broad view of DT usage and its integration with agile methods.

Our future work is the creation of a mechanism to collaborate with the decision making in terms of which techniques to select when using DT, as well as deepening the survey in other communities and other countries besides Brazil.

## Footnotes

1 Available in: https://www.qualtrics.com.

2 Available in: https://www.linkedin.com.

## References

[1] Hehn, J., Uebernickel, F.: The use of design thinking for requirements engineering: an ongoing case study in the field of innovative software-intensive systems. In: Proceedings of International Requirements Engineering Conference (RE), pp. 400–405. IEEE (2018)

[2] Kolko, J.: Design thinking comes of age (2015)

[3] Hiremath M, Sathiyam V, Kotzé P, Marsden G, Lindgaard G, Wesson J, Winckler M (2013). Fast train to DT: a practical guide to coach design thinking in software industry. Human-Computer Interaction – INTERACT 2013.

[4] Liikkanen, A.L., Kilpiö, H., Svan, L., Hiltunen, M.: Lean UX: the next generation of user-centered agile development? In: Proceedings of the Nordic Conference on Human-Computer Interaction, pp. 1095–1100 (2014)

[5] Darrin, M.A.G., Devereux, W.S.: The agile manifesto, design thinking and systems engineering. In: Proceedings of the International Systems Conference, Quebec, Canada, pp. 1–5. IEEE (2017)

[6] Pereira J, Russo R (2018). Design thinking integrated in agile software development: a systematic literature review. Procedia Comput. Sci..

[7] Brown T, Katz B (2011). Change by design. J. Prod. Innov. Manag..

[8] Brown T (2011). Definitions of design thinking. Harv. Bus. Rev..

[9] Vianna M (2012). Design Thinking: inovação em negócios.

[10] Sohaib O, Solanki H, Dhaliwa N, Hussain W, Asif M (2018). Integrating design thinking into extreme programming. J. Ambient Intell. Humaniz. Comput..

[11] Jensen MB, Lozano F, Steinert M, Abrahamsson P, Jedlitschka A, Nguyen Duc A, Felderer M, Amasaki S, Mikkonen T (2016). The origins of design thinking and the relevance in software innovations. Product-Focused Software Process Improvement.

[12] Gurusamy K, Srinivasaraghavan N, Adikari S, Marcus A (2016). An integrated framework for design thinking and agile methods for digital transformation. Design, User Experience, and Usability: Design Thinking and Methods.

[13] Nedeltcheva, G.N., Shoikova, E.: Coupling design thinking, user experience design and agile: towards cooperation framework. In: Proceedings of the International Conference on Big Data and Internet of Thing, pp. 225–229. ACM (2017)

[14] Coutinho, E.F., Gomes, G.A.M., José, M.L.A.: Applying design thinking in disciplines of systems development. In: Proceedings of the Euro American Conference on Telematics and Information Systems, pp. 1–8. EATIS (2016)

[15] De Souza, R.A.C., de Azevedo Cysneiros Filho, G.A., Batista, G.H.C.: A heuristic approach for supporting innovation in requirements engineering. In: Proceedings of the Iberoamerican Conference on Software Engineering, Lima, Peru, p. 674 (2015)

[16] Prasad, W.R., Perera, G., Padmini, K.J., Bandara, H.D.: Adopting design thinking practices to satisfy customer expectations in agile practices: a case from Sri Lankan software development industry. In: Proceedings of the Moratuwa Engineering Research Conference, pp. 471–476. IEEE (2018)

[17] Corral, L., Fronza, I.: Design thinking and agile practices for software engineering: an opportunity for innovation. In: Proceedings of the Annual SIG Conference on Information Technology Education. SIGITE 2018, New York, NY, USA. Association for Computing Machinery, pp. 26–31 (2018)

[18] Rhinow Holger, Meinel Christoph (2013). Design Thinking: Expectations from a Management Perspective. Design Thinking Research.

[19] Nedeltcheva, G.N., Shoikova, E.: Coupling design thinking, user experience design and agile: towards cooperation framework. In: Proceedings of the International Conference on Big Data and Internet of Thing, BDIOT2017, New York, NY, USA. Association for Computing Machinery, pp. 225–229 (2017)

[20] Pfleeger SL, Kitchenham BA (2001). Principles of survey research: part 1: turning lemons into lemonade. ACM SIGSOFT Softw. Eng. Notes.

[21] Kasunic, M.: Designing an effective survey. Technical report, Carnegie-Mellon University Pittsburgh PA Software Engineering Institute (2005)

[22] Kitchenham BA (2002). Preliminary guidelines for empirical research in software engineering. IEEE Trans. Softw. Eng..

[23] Souza, A., Ferreira, B., Conte, T.: Aplicando design thinking em engenharia de software: um mapeamento sistemático. In: Proceedings of the Iberoamerican Conference on Software Engineering, Buenos Aires, Argentina (2017)

[24] Adikari S, McDonald C, Campbell J, Marcus A (2013). Reframed contexts: design thinking for agile user experience design. Design, User Experience, and Usability. Design Philosophy, Methods, and Tools.

[25] Keighran H, Adikari S, Marcus A (2016). Developing high-performing teams: a design thinking led approach. Design, User Experience, and Usability: Design Thinking and Methods.

[26] Mutuku, L.N., Colaco, J.: Increasing Kenyan open data consumption: a design thinking approach. In: Proceedings of the International Conference on Theory and Practice of Electronic Governance, ICEGOV 2012, pp. 18–21. ACM New York (2012)

[27] Glomann L, Ahram T, Falcão C (2018). Introducing ‘human-centered agile workflow’ (HCAW) – an agile conception and development process model. Advances in Usability and User Experience.

[28] Carell A, Lauenroth K, Platz D (2018). Using design thinking for requirements engineering in the context of digitalization and digital transformation: a motivation and an experience report. The Essence of Software Engineering.

[29] Newman, P., Ferrario, M.A., Simm, W., Forshaw, S., Friday, A., Whittle, J.: The role of design thinking and physical prototyping in social software engineering. In: Proceedings of the IEEE International Conference on Software Engineering, vol. 2, pp. 487–496. IEEE/ACM (2015)

[30] Berger, A.: Design thinking for search user interface design. In: Proceedings of the European Workshop on Human-Computer Interaction and Information Retrieval, pp. 1–4 (2011)

[31] Sandino D, Matey LM, Vélez G, Marcus A (2013). Design thinking methodology for the design of interactive real-time applications. Design, User Experience, and Usability. Design Philosophy, Methods, and Tools.

[32] El-Sharkawy S, Schmid K, Berry D, Franch X (2011). A Heuristic approach for supporting product innovation in requirements engineering: a controlled experiment. Requirements Engineering: Foundation for Software Quality.

[33] de Paula DFO, Araújo CC, Stephanidis C (2016). Pet empires: combining design thinking, lean startup and agile to learn from failure and develop a successful game in an undergraduate environment. HCI International 2016 – Posters’ Extended Abstracts.

[34] Lucena P, Braz A, Chicoria A, Tizzei L, Silva da Silva T, Estácio B, Kroll J, Mantovani Fontana R (2017). IBM design thinking software development framework. Agile Methods.

